# From Rights to Risks: Mental Health Implications for Pregnant Patients Following the Reversal of Roe v. Wade

**DOI:** 10.7759/cureus.91886

**Published:** 2025-09-09

**Authors:** Andrew S Murdock, Angelica Arshoun, Eduardo D Espiridion

**Affiliations:** 1 Psychiatry, Drexel University College of Medicine, Philadelphia, USA

**Keywords:** abortion, access to care, laws, marginalized community, psychological impact

## Abstract

Introduction

The overturn of Roe v. Wade threatened reproductive rights, leading to bans, delayed access to care, and potentially increased psychological stress surrounding abortion care. We aim to assess the incidence and prevalence of acute stress episodes (ASE) and depressive episode diagnoses in patients after a non-spontaneous abortion.

Methods

We used a medical record database, TriNetX (TriNetX, LLC, Cambridge, MA), to assess the incidence and prevalence of depressive episodes (International Classification of Diseases, Tenth Revision, Clinical Modification {ICD-10-CM}=F32) and acute stress episodes (ICD-10-CM=F43.0, F43.9, and F43.11) in patients who received non-spontaneous abortions across 66 US healthcare organizations. We used relative risk with 95% confidence intervals to compare outcomes across three periods: pre-COVID (July 2017-2019), COVID (July 2020-2022), and post-Roe v. Wade (pRvW, July 2022-2024). Chi-squared (χ^2^) tests assessed differences within racial groups from 2017 to 2024.

Results

The prevalence of acute psychiatric episodes increased over time (depressive episodes: pre-COVID/COVID=0.779 {0.766-0.791} and COVID/pRvW=0.794 {0.783-0.806}; acute stress episode: pre-COVID/COVID=0.745 {0.713-0.779} and COVID/pRvW=0.739 {0.710-0.770}). The incidence of depressive episodes dipped during COVID and was highest during pRvW (pre-COVID/COVID, 1.062 {1.010-1.117}; COVID/pRvW, 0.845 {0.802-0.890}; pre-COVID/pRvW, 0.897 {0.850-0.947}). The incidence of acute stress episodes increased in the COVID and pRvW periods, but these periods were not statistically different from each other (pre-COVID/COVID, 0.838 {0.741-0.947}; pre-COVID/pRvW, 0.759 {0.667-0.863}). The prevalence of depressive episodes increased significantly for African American patients (χ^2^=15.845, degrees of freedom {dF}=6, and p=0.0146).

Discussion

Overall, there has been an increase in the incidence and prevalence of acute stress episode and depressive episode diagnoses since the overturn of Roe v. Wade, with certain racial groups being disproportionately impacted. This illustrates the need for improved psychological resources, specifically for marginalized communities.

Conclusion

The overturn of Roe v. Wade seems to be associated with an increased incidence and prevalence of acute psychiatric diagnoses after a non-spontaneous abortion. Further studies should be conducted to establish such an association.

## Introduction

Dobbs v. Jackson Women's Health Organization marked a seismic shift in the reproductive rights of millions of Americans. It reversed Roe v. Wade and introduced a new era of legal uncertainty regarding access to care. In the wake of the ruling, many states enacted stringent abortion restrictions, creating significant barriers for those seeking to exercise their reproductive rights [1-3]. Physicians are increasingly worried about the potential legal consequences of aborting a pregnancy, even in situations where the life of the pregnant patient is at risk [3]. In some states, patients are forced to wait until they are on the brink of death before physicians can intervene and provide abortion services, jeopardizing the patient's life and, in some cases, their future ability to reproduce​ [4-6]. Taken together, these barriers cause delays in care, increased travel distances, and, occasionally, the denial of services altogether, which disproportionately affects low-income and marginalized communities who lack the resources to travel out of state to receive care [7-9]. These changes have ignited intense debate and concern over the immediate and long-term effects on patients both physically and psychologically ​[4-6].

In addition to the new logistical and practical challenges to access abortions, these laws may have a profound psychological impact on patients. Previous research demonstrated that access to healthcare is intricately linked to improved mental well-being and decreased mental health crises ​[10-12]. The recent restrictions and looming uncertainties regarding the future of American healthcare further exacerbate patient stress, anxiety, and emotional distress among individuals seeking abortions [13]. These mental health consequences are compounded by the evolving social and economic pressures associated with navigating a newly fragmented and restrictive reproductive healthcare landscape.

This paper examines the psychological implications of restricted abortion access on patients who received non-spontaneous abortions after the reversal decision. Overall, we aim to understand how the hyperdynamic and seemingly volatile legal environment has impacted the mental well-being of this patient population in an effort to offer insights into the broader consequences for public health and policy. We hypothesize that the incidence and prevalence of acute depressive and acute stress episodes (ASE) will increase among pregnant patients who received a non-spontaneous abortion, specifically in the period following June 2022. We also assess the incidence and prevalence during the start of the COVID pandemic, as quarantine policies likely increased stress and isolation. Lastly, we will evaluate the difference in the incidence and prevalence of these acute psychiatric episodes across various racial groups to determine if specific populations are disproportionately affected. The results of our study are necessary to assess gaps in psychiatric care for patients post-abortion.

## Materials and methods

Inclusion and exclusion

We used TriNetX (TriNetX, LLC, Cambridge, MA), a database with de-identified electronic medical records, to assess the incidence and prevalence of acute stress episodes and depressive episode diagnoses among patients who received elective abortions. We limited our search to the US Collaborative Network, which included 66 healthcare organizations. Our cohort included patients aged 18 years or older. Eligible patients received an elective abortion, not spontaneous abortion, indicated by the procedure code Current Procedural Terminology (CPT): 1009003 between July 1, 2017, and June 30, 2024. We used this selected code, CPT: 1009003, to capture surgical interventions to terminate a pregnancy, which includes many methods such as induced abortions via intra-amniotic injections, vaginal suppositories with cervical dilation, dilation and curettage, and dilation and evacuation. Patients were ineligible if their abortion was coded as a spontaneous abortion (International Classification of Diseases, Tenth Revision, Clinical Modification {ICD-10-CM}: O03). We did not include emergency nonelective abortions such as those performed because of maternal risk. Patients of all racial and ethnic backgrounds were included in the study, and patients were not excluded if they identified with more than one racial group. Data was pulled and analyzed on March 2, 2025. In the analysis from this cohort (N=113847), we assessed two events of interest: patients with a diagnosis related to a depressive episode (n=9963) or an acute stress episode (ASE, n=1788). Our analysis used a look-back period of one day before the start of each time window to count patients. Eligible patients were counted as either experiencing an acute stress or depressive episode if they had a diagnosis indicated in Table 1.

**Table 1:** Defining events of interest and diagnosis codes for each event of interest are listed. ICD-10-CM: International Classification of Diseases, Tenth Revision, Clinical Modification

Diagnosis Code (ICD-10-CM)	Description
Acute stress episode	
F43.0	Acute stress reaction
F43.9	Reaction to severe stress, unspecified
F43.11	Post-traumatic stress disorder, acute
Depressive episode	
F32	Depressive episode
F32.0	Major depressive disorder, single episode, mild
F32.1	Major depressive disorder, single episode, moderate
F32.2	Major depressive disorder, single episode, severe without psychotic features
F32.3	Major depressive disorder, single episode, severe with psychotic features
F32.4	Major depressive disorder, single episode, in partial remission
F32.5	Major depressive disorder, single episode, in full remission
F32.8	Other depressive episodes
F32.9	Major depressive disorder, single episode, unspecified

Three time periods were assessed: pre-COVID (July 1, 2017, to June 30, 2019), COVID (July 1, 2020, to June 30, 2022), and post-Roe v. Wade (pRvW) (July 1, 2022, to June 30, 2024). The July 1 cutoff was selected due to the Roe v. Wade decision being made at the end of June 2022. Risk ratios with associated 95% confidence intervals and p-values (α=0.05) were calculated to assess differences in the incidence and prevalence of acute stress and depressive episode diagnoses across the periods. The data was then stratified by race, and trends for each subgroup from July 1, 2017, to June 30, 2024, were evaluated with multiple chi-squared (χ^2^) tests (α=0.05). Patients who identified as more than one race were in each racial category they selected. This did not change the total (N).

This retrospective study is exempt from informed consent. The data reviewed is a secondary analysis of existing data, does not involve intervention or interaction with human subjects, and is de-identified per the de-identification standard defined in Section 164.514(a) of the Health Insurance Portability and Accountability Act of 1996 (HIPAA) Privacy Rule. The process by which the data is de-identified is attested to through a formal determination by a qualified expert as defined in Section 164.514(b)(1) of the HIPAA Privacy Rule. This formal determination by a qualified expert was refreshed in December 2020.

## Results

Out of 113847 eligible patients during the study period, 9963 (8.75%) and 1788 (1.57%) were diagnosed with depressive episodes and acute stress episodes, respectively (Table 2). The "male" and "unknown" categories were included as gender was not an exclusion criterion and also to be inclusive of the lesbian, gay, bisexual, transgender, and queer (LGBTQ) community.

**Table 2:** Demographic characteristics.

	Total		Depressive Episode		Acute Stress Episode	
	N	%	N	%	N	%
N	113847	100	9963	8.75	1788	1.57
Sex						
Female	110671	97.21				
Unknown	3131	2.75				
Male	46	0.04				
Race						
White	59178	51.98	5691	57.12	791	44.24
Unknown race	20447	17.96	1437	14.42	250	13.98
Black or African American	19365	17.01	1958	19.65	509	28.47
Other race	7093	6.23	460	4.62	85	4.75
Asian	6603	5.80	268	2.69	73	4.08
Native Hawaiian or other Pacific Islander	729	0.64	79	0.79	50	2.80
American Indian or Alaska Native	433	0.38	70	0.70	30	1.68

Overall, the analysis of incidence and prevalence shows an increasing proportion over the periods assessed (Table 3).

**Table 3:** Incidence and prevalence of depressive and acute stress episodes pre-COVID, during COVID, and post-Roe v. Wade (pRvW)

	Depressive Episode				Acute Stress Episode			
	Incidence		Prevalence		Incidence		Prevalence	
Period	%	n	%	n	%	n	%	n
Pre-COVID	2.33	3205	15.99	22647	0.31	476	2.39	3399
COVID	2.25	2846	18.65	28357	0.37	551	2.92	4446
pRvW	2.66	2393	23.48	26834	0.41	453	3.96	4521

**Figure 1:**
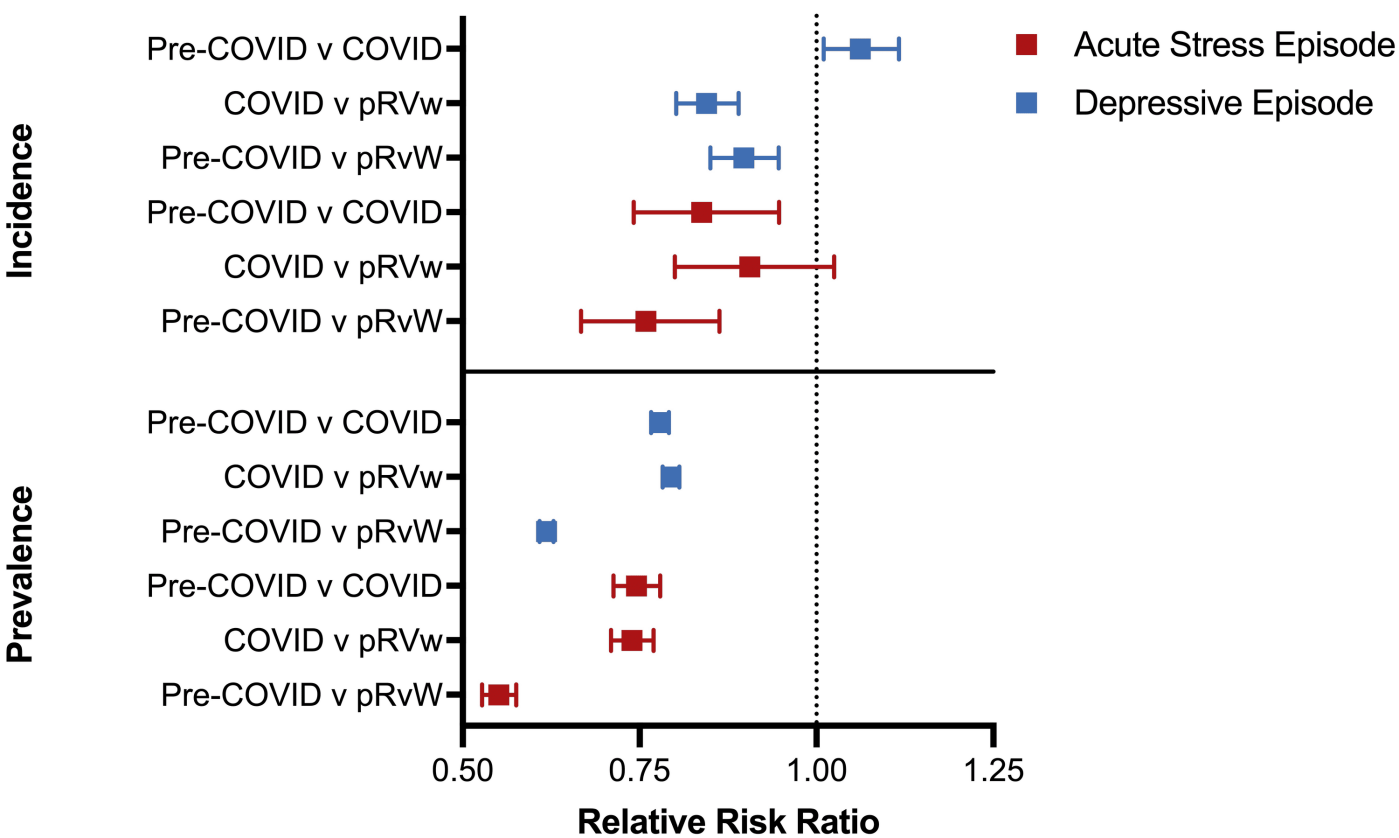
Relative risk comparison of the incidence and prevalence of acute psychiatric diagnoses between different figures. pRvW: post-Roe v. Wade

Incidence trends across periods were different for depressive and acute stress episodes, but there was no change in the prevalence trends (Figure 1). For depressive episodes, the incidence in the pre-COVID period was significantly greater than the COVID period, but it was significantly lower than the pRvW period (Figure 1; pre-COVID/COVID, 1.062 {1.010-1.117}; COVID/pRvW, 0.845 {0.802-0.890}; pre-COVID/pRvW, 0.897 {0.850-0.947}). The incidence of acute stress episodes was significantly lower in the pre-COVID period compared to the other periods, but the difference between the COVID and pRvW periods was insignificant (Figure 1; pre-COVID/COVID, 0.838 {0.741-0.947}; COVID/pRvW, 0.906 {0.800-1.025}; pre-COVID/pRvW, 0.759 {0.667-0.863}). 

**Figure 2:**
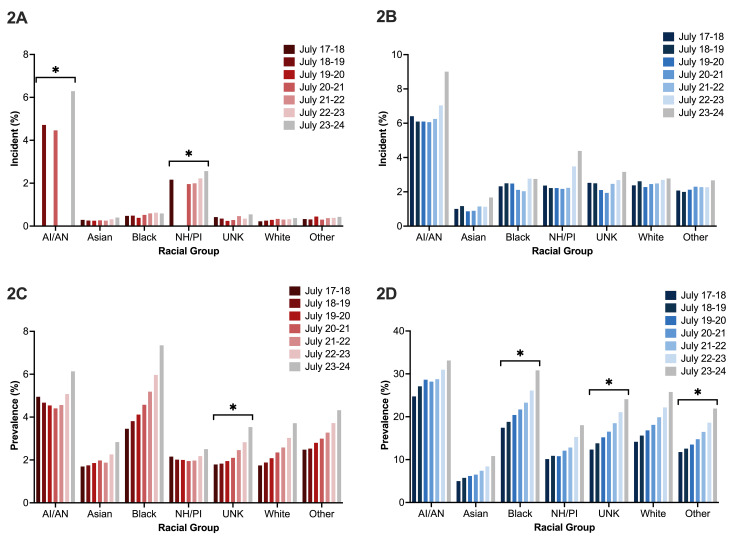
Assessing the incidence and prevalence of acute stress post-non-spontaneous abortions across racial groups from July 2019 to July 2024. The incidence and prevalence of acute stress episodes are indicated in A and C, respectively. The incidence and prevalence of depressive episodes are indicated in B and D, respectively. Statistical significance was calculated using chi-squared tests. *Groups where a statistically significant change was noted (p≤0.05). AI/AN, American Indian or Alaska Native; NH/PI, Native Hawaiian or Pacific Islander; UNK, unknown

Chi-squared analysis showed that certain racial groups had significant differences in the prevalence of acute stress episode and depressive episode diagnoses between 2017 and 2024. A significant difference between the observed and expected prevalence of acute stress episodes was only present in those identifying as "American Indian or Alaska Native" and "Hawaiian or other Pacific Islander" (Figure 2A; degrees of freedom {dF}=6; American Indian or Alaska Native: χ^2^=40 and p<0.0001; Native Hawaiian or other Pacific Islander: χ^2^=20.238, p=0.00250, and dF=6; Asian: χ^2^=40 and p=0.994; Black or African American: χ^2^=2.453 and p=0.864; unknown race: χ^2^=10.807 and p=0.0945; White: χ^2^=6.994 and p=0.321; other race: χ^2^=0.776 and p=0.996). These groups had certain years with zero incidence (Figure 2A). For the prevalence of acute psychiatric episodes, there was only a significant increase from expected values for those who identified as "unknown race" (Figure 2C; dF=6; American Indian or Alaska Native: χ^2^=1.237 and p=0.977; Asian: χ^2^=2.673 and p=0.268; Black or African American: χ^2^=3.131 and p=0.0146; Native Hawaiian or other Pacific Islander: χ^2^=1.237 and p=0.574; unknown: χ^2^=13.335 and p≤0.0001; White: χ^2^=1.175 and p=1; other race: χ^2^=0.204 and p=0.00360). 

The analysis of depressive episodes shows no significant difference in the prevalence of depressive episodes across the racial groups (Figure 2B; dF=6; American Indian or Alaska Native: χ^2^=1.91 and p=0.977; Asian: χ^2^=7.616 and p=0.268; Black or African American: χ^2^=9.917 and p=0.128; Native Hawaiian or other Pacific Islander: χ^2^ = 4.767 and p=0.574; unknown race: χ^2^=9.943 and p=0.127; White: χ^2^=3.02 and p=0.999; other race: χ^2^=6.707 and p=0.349). The prevalence of depressive episodes was significantly higher than expected values for "Black or African American," "unknown race," and "other race" groups (Figure 2D; dF=6; Black or African American: χ^2^=15.845 and p=0.015; unknown race: χ^2^=75.634 and p<0.001; other: χ^2^=19.368 and p=0.004). Other racial groups had no significant change in the expected incidence or prevalence of depressive episodes (Figure 2B; dF=6; American Indian or Alaska Native: χ^2^=1.91 and p=0.977; Asian: χ^2^=7.616 and p=0.268; Native Hawaiian or other Pacific Islander: χ^2^=4.767 and p=0.574; White: χ^2^=0.302 and p=0.999).

The prevalence of both acute psychiatric conditions significantly increased over time, with the peak prevalence in the pRvW period (Figure 1; depressive episode: pre-COVID/COVID=0.779 {0.766-0.791}, COVID/pRvW=0.794 {0.783-0.806}, and pre-COVID/pRvW=0.619 {0.609-0.629}; acute stress episode: pre-COVID/COVID=0.745 {0.713-0.779}, COVID/pRvW=0.739 {0.710-0.770}, and pre-COVID/pRvW=0.551 {0.527-0.576}).

## Discussion

This study explored the incidence and prevalence of depressive and acute stress episodes before and after important nationwide events: pre-COVID (2017-2019), the immediate COVID years (2020-2022), and the years following the overturning of Roe v. Wade (2022-2024). The incidence of depressive episodes dipped during the COVID period, potentially due to decreased access to medical and psychiatric care secondary to in-person and financial constraints during the pandemic [14]. Less access causes fewer diagnoses, which may explain this finding. During COVID, many doctors' offices began implementing virtual options for their patients and maintained these options even as pandemic-related restrictions were removed [15]. This could have led to a subsequent increase in the number of patients who were able to access psychiatric care in the pRvW period, as doctors' appointments were now more accessible, economic burdens were lessened, and there was heightened awareness regarding the importance of social support and mental well-being. For acute stress episodes, a rise in incidence occurred in the COVID era and remained in pRvW. While we cannot account for episode severity, it is possible that the acute stress reactions were more severe during COVID than the depressive episodes, therefore warranting clinical attention.

The increased prevalence of both psychiatric outcomes over time suggests opportunities for clinical intervention. While the prevalence of acute stress episodes was less than 4%, our data indicates that about one in four patients had a depressive episode at some point after their non-spontaneous abortion post-Roe v. Wade. It is possible that the increase in acute psychiatric diagnoses is caused by growing concerns due to nationwide events or increasing stigma surrounding abortion care. This indicates a potential need for psychiatric follow-up after a non-spontaneous abortion.

Specific minority populations experienced a disproportionate increase in mental health diagnoses. It is well established that the intersectionality between identifying as a racial minority and a woman leads to compounded experiences of societal inequities​ [16]​. The intersectionality theory would lead one to believe that there would be heightened rates of psychiatric episodes across all minorities; however, our data indicates that specific racial groups may be particularly susceptible to developing psychiatric episodes after an abortion.

For depressive episodes, "Black and African American" patients, patients whose race was unknown or not disclosed, and patients who self-identified as "other" had statistically significant increases in the prevalence of new depressive episodes compared to the other populations studied (Figure 2). The elevated rates of depression observed among Black patients with non-spontaneous abortions highlight a critical area for intervention. Such findings align with existing literature suggesting that racial and systemic factors contribute significantly to mental health disparities, specifically that marginalized groups often experience higher levels of mental health issues due to systemic inequalities and stressors​ [17-19]. The statistically significant increases within these two ambiguous racial categories may result from an increased number of patients attempting to hide some aspect of their identity for their own protection. Alternatively, an increased number of individuals who do not identify as any of the races defined by the US Census may feel inclined to self-report "other." Additional studies are necessary to determine if either case is true.

For acute stress episodes, the significantly elevated incidence and prevalence in Indigenous populations might indicate another area for intervention. Historically, Indigenous communities faced a range of unique stressors, including intergenerational trauma, cultural dislocation, and ongoing social inequities ​[20]. These factors may contribute to a heightened vulnerability to anxiety following a non-spontaneous abortion. However, our findings are complicated by the fact that certain years have no record of Indigenous patients with acute stress diagnosis codes. Reasons for this finding could include a small sample size, the lack of access to mental health care, or patients seeking care at hospitals not covered in our analysis. Overall, our findings underscore the need for culturally sensitive mental health support tailored to Indigenous individuals' specific experiences and challenges and improved access for these populations.

This study has inherent limitations based on its design. Assessing ICD-10 codes is a convenient way to gather copious amounts of data in a brief period, but multiple factors can impact the accuracy of the data. First, different providers may select differing billing codes for similar diagnoses. Second, the present cohort does not encompass patients who did not or could not access the US healthcare system for their abortion or for their mental health care. Multiple barriers, including insurance status, distance from the nearest clinic, the stigma surrounding the procedure, and even childcare needs for some patients, could prevent patients from accessing an abortion. This includes people in states that have banned or limited abortion access who sought "unsafe abortions," defined as an abortion performed by individuals who lack necessary skills, lack proper medical equipment, or both ​[21]. While this population is difficult to assess with billing codes, studies have shown that restricting abortion access increases these practices​ [16]. Third, subjects with psychiatric episodes may not have sought mental health care following their abortion for various reasons, thereby underestimating the psychiatric burden of non-spontaneous abortions. Reasons could include increased stigma surrounding abortions, changing work dynamics during and after the pandemic, and exacerbated economic challenges. Finally, we could not separate multiracial individuals into unique categories when assessing differences among races. More comprehensive reporting systems may be needed to establish differences. These limitations in the TriNetX platform are important to consider since some were not possible to control, such as balancing for the state or region. Issues such as socioeconomic status, health insurance, and prior psychiatric history will provide a better picture of the current situation.

Future research could assess if the overturning of Roe v. Wade has impacted the comorbidity rate of other acute psychiatric conditions in people who received non-spontaneous abortions, such as new-onset substance use or substance use relapse. Alternatively, one could assess psychiatric comorbidities in patients with spontaneous abortions. An exploration of nonpsychological complications, such as sepsis or fertility issues, could further illustrate the health impact the Supreme Court's decision has had on patients receiving an abortion. Future studies should also use more inclusive categories for assessing racial groups, as more comprehensive racial categories may provide clearer information regarding the lived experiences of people from a wide variety of backgrounds and ethnicities. Some recommendations, such as integrating routine mental health screening post-abortion and ensuring culturally competent support services for Indigenous, African American, and other disproportionately affected groups, will give a clearer picture of this situation. In the meantime, physicians should be aware that certain patients who receive non-spontaneous abortions may now be at an increased risk of developing acute psychological complications following their procedure. There are also important issues that could affect the outcome for these patients, including routine mental health screening in post-abortion follow-up, as well as improving social and economic conditions related to it. Health access is also a very important issue that could impact the outcome.

## Conclusions

The overturn of Roe v. Wade seems to be associated with an increased incidence and prevalence of acute stress episode and depressive episode diagnoses after a non-spontaneous abortion. Further studies should be conducted to establish the association. Analysis across racial subgroups indicated that African American patients had an increased prevalence of depressive episodes from 2017 to 2024. We also found an increased prevalence of acute stress and depressive episode diagnoses in patients identifying as unknown and an increased prevalence of depressive episodes in those identifying as other, potentially indicating that patients are seeking anonymity in their abortion or mental health care. Ultimately, these associations demonstrate a need for patients to receive psychiatric follow-up after a non-spontaneous abortion.
